# The Association Between Meteorological Factors and Rotavirus Gastroenteritis Incidence in Japan: A Time-Series Analysis

**DOI:** 10.7759/cureus.89675

**Published:** 2025-08-09

**Authors:** Keita Wagatsuma

**Affiliations:** 1 Division of International Health, Graduate School of Medical and Dental Sciences, Niigata University, Niigata, JPN

**Keywords:** association, attributable fraction, climate, japan, rotavirus

## Abstract

Introduction

Rotavirus is the principal pathogen responsible for acute gastroenteritis and severe diarrhea in children worldwide and remains a significant public health threat. However, studies on the association between rotavirus gastroenteritis epidemics and meteorological factors in Japan are still scarce. In this study, we aimed to quantify the short-term effects of meteorological factors on the incidence of rotavirus gastroenteritis in Japan using advanced time-series modeling approaches.

Methods

We conducted a two-stage time-series analysis. In the first stage, a time-stratified case-crossover study was performed using conditional quasi-Poisson regression in conjunction with a distributed lag nonlinear model to assess the association between meteorological factors and the incidence of rotavirus gastroenteritis across all 47 Japanese prefectures from 2014 to 2019. A multivariate meta-analysis was subsequently used to pool associations at the national level.

Results

A total of 26,549 cases of rotavirus gastroenteritis were included in the study. Our analysis revealed that low mean temperature and reduced relative humidity were significantly associated with an increased incidence of rotavirus gastroenteritis. More specifically, the overall lag-cumulative relative risk (RR) of rotavirus gastroenteritis peaked at approximately 13.0°C (RR: 2.2, 95% confidence interval (CI): 1.9-2.6) relative to a reference mean temperature of 25°C. Similarly, the RR was maximized at a relative humidity of approximately 55% (RR = 1.7, 95% CI: 1.4-2.2) compared with 77%.

Conclusions

This nationwide study showed that cold and dry environmental conditions were associated with an elevated risk of rotavirus gastroenteritis, which varied markedly based on region.

## Introduction

Rotavirus is the principal pathogen responsible for acute gastroenteritis and severe diarrhea in children worldwide and remains a significant public health threat [1]. Recent evidence from the Global Burden of Disease 2019 Study, which evaluated data spanning approximately 30 years, indicated that rotavirus accounted for 19.11% of all deaths due to diarrheal disease in 2019 [2]. Extensive epidemiological studies have unequivocally shown that environmental determinants contribute substantially to seasonal fluctuations in rotavirus infections [3].

Typically, rotaviruses exhibit marked seasonality, with their incidence peaking from winter to early spring in countries such as Argentina and Japan [4,5]. Observational studies have revealed that low ambient temperature and reduced relative humidity are associated with an elevated risk of rotavirus disease [6]. However, previous epidemiological studies have predominantly focused on the short-term effects of meteorological factors within individual regions or cities, with insufficient quantification of these effects in multisite settings, leading to considerable inconsistencies across different locations in the extant literature [7,8].

In this study, we aimed to address these research gaps by assessing the short-term effects of meteorological factors on the incidence of rotavirus gastroenteritis, quantified in terms of relative risk (RR), using data from a six‐year follow‐up period across all 47 prefectures in Japan.

## Materials and methods

Data collection

We obtained national surveillance data on weekly notifications of rotavirus gastroenteritis cases from 47 Japanese prefectures between 2014 and 2019 [9]. These data were sourced from the Infectious Disease Weekly Report compiled by the National Institute of Infectious Diseases, which conducts nationwide surveillance via a network of approximately 3,000 designated pediatric sentinel sites, including hospitals and clinics with pediatric departments. Ambient meteorological data, including daily mean temperature (°C), relative humidity (%), wind speed (m/s), and sunshine duration (h), were obtained from the Japan Meteorological Agency [10]. Measurements were recorded at meteorological monitoring stations located in the capital city of each prefecture and were subsequently aggregated into weekly averages. 

This study was based exclusively on publicly available, aggregated datasets that had been de-identified and fully anonymized before analysis. Secondary analyses of such anonymized, aggregate data are not classified as human-subjects research. Consequently, approval by an institutional ethics committee was not required, and the requirement to obtain individual informed consent was waived. The study was conducted per the Declaration of Helsinki.

Statistical analysis

A two-stage design was employed [11]. In the first stage, we examined the relationship between meteorological factors and the incidence of rotavirus gastroenteritis at the national level and within 47 prefectures. This was achieved through a time-stratified case-crossover analysis that integrated conditional quasi-Poisson regression with distributed lag nonlinear models [12]. Time stratification was implemented using a two-way interaction between years and months. Within the distributed lag nonlinear model framework, we employed a cross‐basis function to accommodate nonlinear exposure-response relationships as well as multi‐lagged effects. Specifically, the exposure-response association was modelled using a natural cubic B‐spline with knots positioned at the 25^th^, 50^th^, and 75^th^ percentiles of the mean temperature and relative humidity, whereas the lag-response relationship was represented by a natural cubic B‐spline over a five‐week period, with three equidistant knots on a logarithmic scale. Additionally, potential confounders, namely, wind speed and sunshine duration, were adjusted using a natural cubic B-spline with three degrees of freedom.

Further adjustments were made to the number of public holidays per week, and an autoregressive term for the logarithm of weekly rotavirus gastroenteritis cases (with a one-week lag) was incorporated [13]. In the second stage, a fixed-effects (intercept‐only) meta-analysis was performed to pool the prefecture-specific estimates derived in the first stage, thereby yielding a nationwide association [14]. Residual heterogeneity was evaluated using Cochran’s Q test, and interprefectural variability was quantified using the I² statistic. Overall lag‐cumulative RRs and corresponding 95% confidence intervals (CIs) were estimated with reference to the minimum morbidity temperature (MMT) and minimum morbidity humidity (MMH), defined as the mean temperature and relative humidity at which the incidence of rotavirus gastroenteritis was minimized, respectively.

Sensitivity analyses were performed to assess the robustness of the findings. First, the placement of knots in the exposure-response function varied across different configurations, specifically at the 25^th^, 50^th^, and 75^th^ percentiles; 10th, 50th, and 90th percentiles; and 10^th^, 75^th^, and 90^th^ percentiles. Second, considering the incubation period, the maximum lag period was extended from four to six weeks to evaluate the stability of the observed associations. Statistical significance was determined at two-tailed p < 0.05. All analyses were conducted using R (version 4.1.0; R Foundation for Statistical Computing, Vienna, Austria), utilizing the gnm, dlnm, attdl, and mixmeta packages.

## Results

Descriptive statistics

**Figure 1:**
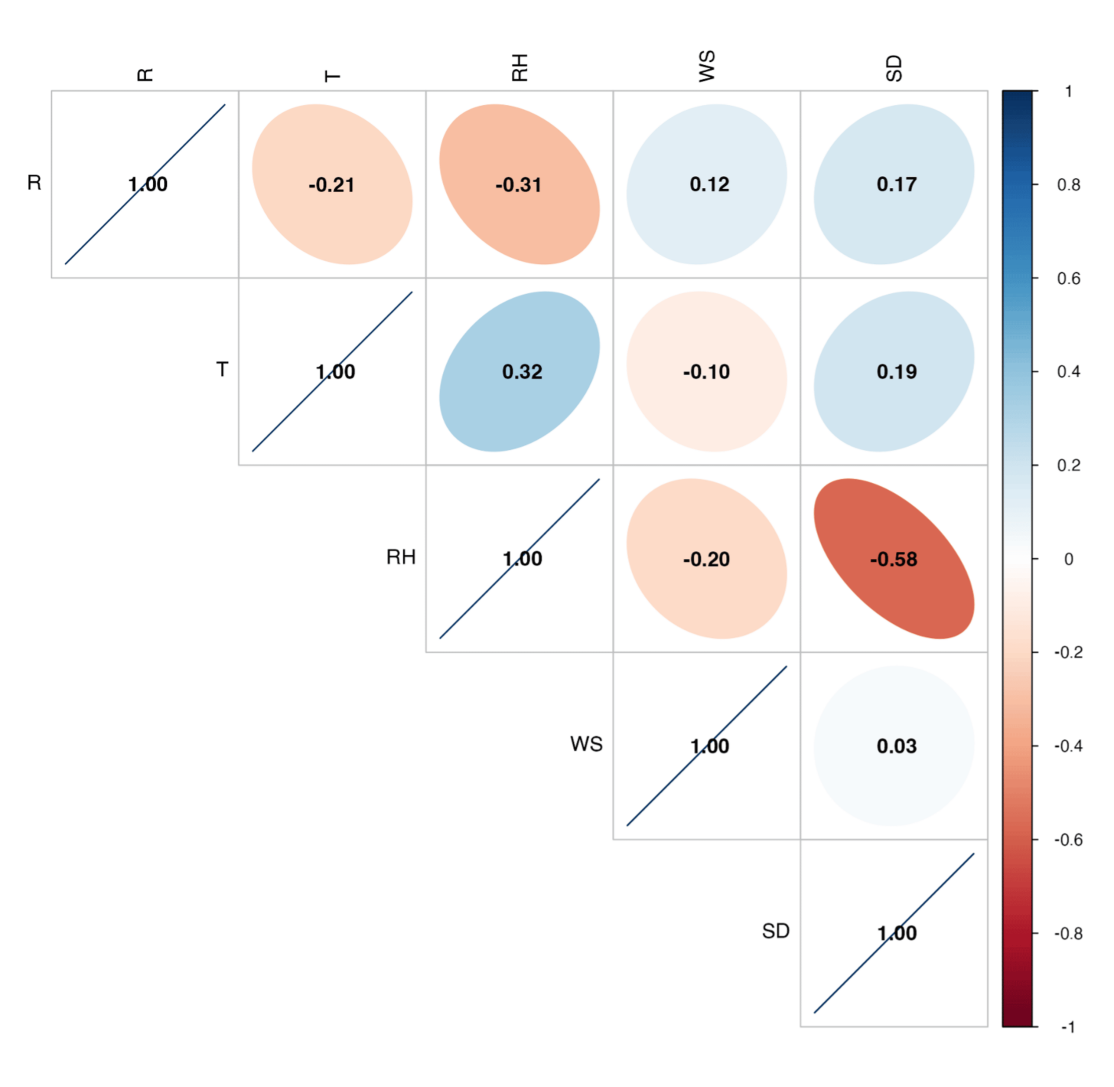
Spearman’s rank-order correlation coefficients among the weekly distributions of rotavirus gastroenteritis incidence and meteorological factors in Japan between 2014 and 2019 R: number of rotavirus gastroenteritis cases; T: mean temperature; RH: relative humidity; WS: wind speed; SD: sunshine duration

Between 2014 and 2019, 26,549 cases of rotavirus gastroenteritis were reported in Japan (Table 1). The nationwide mean weekly case count was 2.0 (standard deviation (SD) = 3.9). Over the study period, the mean weekly temperature was 16.0°C (SD = 8.4°C), and the mean weekly relative humidity was 70.0% (SD = 9.3%). Furthermore, the number of rotavirus gastroenteritis cases was negatively correlated with both mean temperature and relative humidity, with Spearman’s rank-order correlation coefficients of -0.21 and -0.31, respectively (Figure 1).

**Table 1:** Descriptive statistics for rotavirus gastroenteritis cases and meteorological factors between 2014 and 2019 in Japan Summary statistics were calculated based on the weekly prefecture-level averages of each variable SD: standard deviation; Q1: 25^th^ percentile; Q3: 75^th^ percentile

Variable	Total	Mean	SD	Minimum	Q1	Median	Q3	Maximum
Number of rotavirus gastroenteritis cases	26,549	2.0	3.9	0.0	0.0	0.0	2.0	57.0
Mean temperature (°C)	-	16.0	8.4	−7.0	9.0	17.0	23.0	32.0
Relative humidity (%)	-	70.0	9.3	31.0	64.0	71.0	77.0	97.0
Wind speed (m/s)	-	2.9	0.9	0.9	2.2	2.8	3.4	11.7
Sunshine duration (h)	-	5.4	2.3	0.0	3.8	5.4	6.9	12.9

Results of primary analysis

**Figure 2:**
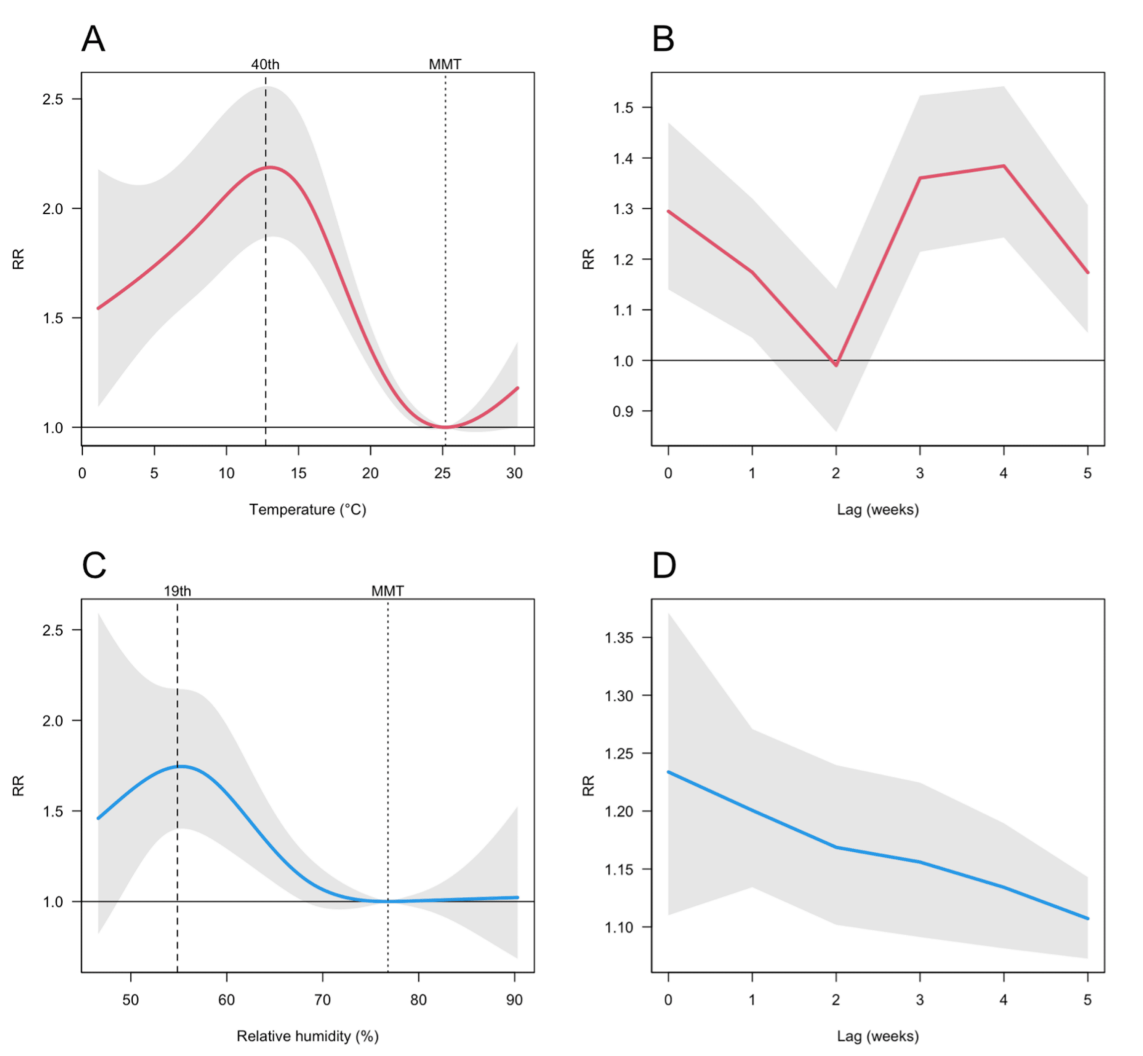
Associations between meteorological factors and the incidence of rotavirus gastroenteritis in Japan (A) Overall lag-cumulative association between the mean temperature (red line) and rotavirus gastroenteritis incidence, with the shaded area representing the 95% CI. Vertical dotted lines denote the 40^th^ percentile (13°C) and minimum morbidity temperature (25 °C) for the mean temperature. (B) Lag–response association between the mean temperature (red line) and rotavirus gastroenteritis incidence at the 40^th^ percentile (13°C), with the corresponding 95% CI illustrated as a shaded area. (C) Overall lag‐cumulative association between relative humidity (blue line) and rotavirus gastroenteritis incidence, with the 95% CI depicted as a shaded area. Vertical dotted lines indicate the 19^th^ percentile (55.0%) and minimum morbidity humidity (77.0%) for relative humidity. (D) Lag–response association between relative humidity (blue line) and rotavirus gastroenteritis incidence at the 19^th^ percentile (55.0%), with the 95% CI shown as a shaded area RR: relative risk; CI: confidence interval; MMT: minimum morbidity temperature; MMH: minimum morbidity humidity

Overall, the pooled lag‐cumulative associations for mean temperature and relative humidity revealed distinct nonlinear patterns (Figures 2A and 2C). In general, low mean temperatures and reduced relative humidity were associated with an increased RR relative to MMT and MMH, respectively. Specifically, with regard to mean temperature, the risk of rotavirus gastroenteritis peaked at approximately 13.0°C (RR = 2.2, 95% CI: 1.9-2.6) compared with an MMT of 25.0°C. Beyond this threshold, RR gradually declined, forming an inverted U-shaped relationship. Similarly, for relative humidity, the risk reached its maximum at approximately 55.0% (RR = 1.7, 95% CI: 1.4-2.2) relative to an MMH of 77.0%. Figures 2B and 2D further depict the lag-response relationships: for mean temperature, the RR peaked immediately after exposure (week 0) and again during the latter part of week four, whereas for relative humidity, the RR significantly peaked at week 0 and subsequently declined over the following weeks. Overall, the meta-analysis revealed significant geographical heterogeneity, with the Q-value of 352.0 (p < 0.001, I² = 47.7%) and 381.6 (p < 0.001, I² = 51.8%) for the mean temperature and relative humidity, respectively.

Sensitivity analyses confirmed that these associations remained largely consistent following adjustments for parameters, such as knot placement and lag structure, thereby highlighting the robustness of our findings (Figure 4).

**Figure 3:**
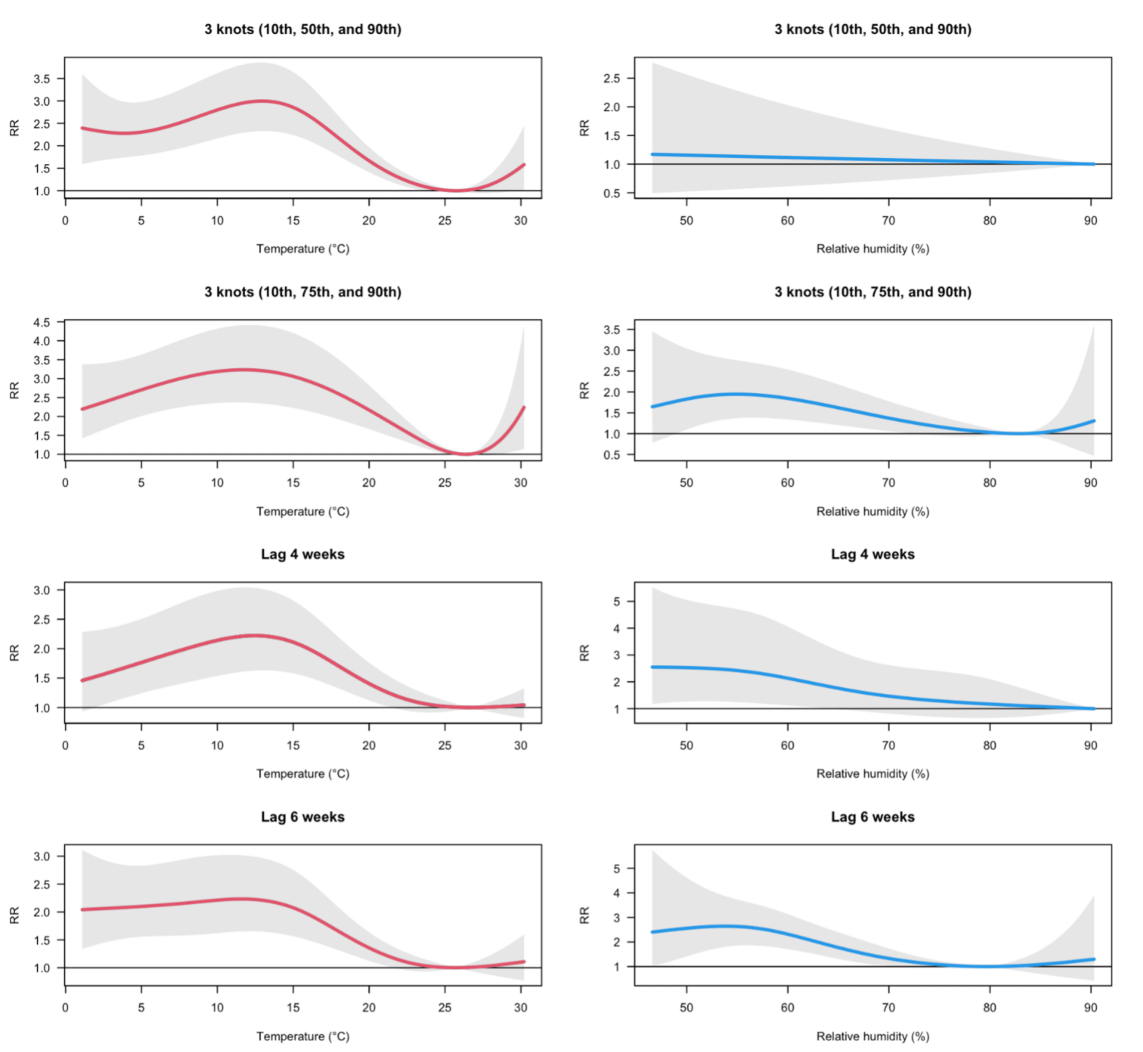
Sensitivity analyses of the overall lag-cumulative associations between meteorological factors and rotavirus gastroenteritis incidence in Japan between 2014 and 2019 The analyses were performed by (i) altering the knot placements for seasonal adjustment—from the default 25^th^, 50^th^, and 75^th^ percentiles to alternative configurations (10^th^, 50^th^, and 90^th^ or 10^th^, 75^th^, and 90^th^ percentiles), and (ii) modifying the lag period from 5 weeks to 4 or 6 weeks RR: relative risk

## Discussion

Our findings provide robust evidence that low mean temperature and reduced relative humidity are associated with an increased risk of rotavirus gastroenteritis in Japan. These results further reinforce the existing epidemiological evidence by comprehensively modelling the exposure-lag-response relationships between rotavirus gastroenteritis cases and meteorological factors. In our study, low mean temperatures and reduced relative humidity were linked to an increased number of cases, a finding that corroborates the results of a previous study showing that the risk of rotavirus infection declines as ambient temperature rises [15]. Laboratory studies have similarly shown that both the viability and infectivity of rotaviruses diminish markedly with increasing temperature [16]. Under cooler conditions, viral particles exhibit enhanced stability and prolonged persistence on human hands, in excreta, and on contaminated surfaces, thereby potentially facilitating fecal-oral transmission [17].

Colder environments tend to promote increased indoor congregation, thereby elevating the risk of exposure to infected individuals as well as to contaminated surfaces, air, food, or water, particularly among vulnerable populations [17]. In terms of relative humidity, a meta-analysis of epidemiological studies from tropical regions reported that a 1% increase in relative humidity was associated with a 3% reduction in the incidence of rotavirus infection, lending further support to our findings [18]. Under relatively low-humidity conditions, rotaviruses may also be prone to aerosolization, thereby increasing the likelihood of transmission via the respiratory route [19]. Although the association between the mean temperature and rotavirus gastroenteritis has been extensively examined, the relationship between relative humidity and rotavirus gastroenteritis remains largely unexplored.

To our knowledge, this investigation represents the first nationwide analysis in Japan to employ a cutting‐edge time‐series modelling approach to elucidate the exposure-lag-response relationships between meteorological factors and the incidence of rotavirus gastroenteritis across all 47 prefectures. A time-stratified case-crossover design was implemented, conferring several methodological advantages, including independence from sample size constraints and inherent control of temporal confounders, thereby effectively accounting for both long-term and seasonal trends.

Nevertheless, this study has several limitations. First, the time-series analysis was conducted within an ecological framework that inherently restricted the capacity for causal inference. Second, this study was confined to Japan, thereby limiting the generalizability of the findings to other regions or countries. Finally, the analysis did not incorporate vaccines into immunization rates, individual-level exposure, or other key potential confounders, such as age, sex, socioeconomic status, and additional environmental determinants. Several methodological approaches have also been proposed for modelling the associations between meteorological factors and health outcomes, including traditional statistical models as well as more recent artificial intelligence-based methods (e.g., machine learning) [20]. Such diversity in modelling strategies may, in part, explain the variability in reported associations across studies.

## Conclusions

This nationwide study indicates that cold and dry conditions may exacerbate the risk of rotavirus gastroenteritis in Japan. These findings provide robust epidemiological evidence that can underpin the development of targeted strategies for preventing and controlling rotavirus infections, ultimately mitigating the overall disease burden. Further investigation is warranted to elucidate the mechanisms underlying this association.
